# The use of social features in mobile health interventions to promote physical activity: a systematic review

**DOI:** 10.1038/s41746-018-0051-3

**Published:** 2018-09-04

**Authors:** Huong Ly Tong, Liliana Laranjo

**Affiliations:** Centre for Health Informatics, Australian Institute of Health Innovation, Macquarie University, Sydney, NSW Australia

**Keywords:** Public health, Lifestyle modification

## Abstract

Mobile health (mHealth) technologies have increasingly been used in interventions to promote physical activity (PA), yet, they often have high attrition rates. Integrating social features into mHealth has the potential to engage users; however, little is known about the efficacy and user engagement of such interventions. Thus, the aim of this systematic review was to characterize and evaluate the impact of interventions integrating social features in mHealth interventions to promote PA. During database screening, studies were included if they involved people who were exposed to a mHealth intervention with social features, to promote PA. We conducted a narrative synthesis of included studies and a meta-analysis of randomized controlled trials (RCTs). Nineteen studies were included: 4 RCTs, 10 quasi-experimental, and 5 non-experimental studies. Most experimental studies had retention rates above 80%, except two. Social features were often used to provide social support or comparison. The meta-analysis found a non-significant effect on PA outcomes [standardized difference in means = 0.957, 95% confidence interval −1.09 to 3.00]. Users’ preferences of social features were mixed: some felt more motivated by social support and competition, while others expressed concerns about comparison, indicating that a one-size-fits-all approach is insufficient. In summary, this is an emerging area of research, with limited evidence suggesting that social features may increase user engagement. However, due to the quasi-experimental and multi-component nature of most studies, it is difficult to determine the specific impact of social features, suggesting the need for more robust studies to assess the impact of different intervention components.

## Introduction

Regular physical activity (PA) is associated with many physical and mental health benefits. Previous studies have demonstrated that PA can be effective in the prevention and treatment of a wide range of diseases, such as hypertension, stroke, type 2 diabetes, several types of cancer, depression, and anxiety.^1–3^ The World Health Organization recommends that adults should do at least 150 min of moderate intensity or 75 min of vigorous intensity PA, throughout 1 week.^4^ Notably, there is a dose-response relationship between PA and cardiovascular outcomes, with higher levels of PA leading to greater health benefits.^5^ Despite the importance of PA, a third of adults and four-fifths of adolescents worldwide fail to meet the recommended levels of PA.^6^ This highlights the importance of finding effective ways to promote PA to reduce morbidity and mortality, as well as health care costs.

The growing availability of mobile health (mHealth) technologies, such as activity trackers or mobile applications (apps) has given rise to new opportunities to influence PA behavior. Specifically, they can be used by individuals at any time and in any environment, enabling the collection of objective, reliable data on PA measures.^7,8^ mHealth technology is increasingly being used in PA interventions, with encouraging results.^9^ However, so far, these interventions have not been adopted by large number of users and often have high attrition rates.^10^ A meta-analysis has found that online social networks (OSNs) can improve intervention retention rates, as well as have a significant positive effect on health behavior change.^11^ Thus, integrating some social features from OSNs (e.g., social support, social comparison) into mHealth technologies could help engage users and result in positive health outcomes.

Several systematic reviews examined the use of mHealth technologies to promote PA, but they were often limited to a single mode of mHealth technology, or a specific setting.^12–18^ No systematic review has examined the use of social features across mobile apps or wearable PA trackers, which limits the ability of researchers and developers to assess the impact of such features on efficacy and user engagement. Thus, the aim of this study was to characterize the use of social features in mobile health (mHealth) interventions to promote physical activity, as well as their effectiveness and impact on users’ preferences and engagement. Specifically, our research questions were:

1. What are the characteristics and effectiveness of mobile health interventions with social features in promoting PA, for both patients and healthy consumers?
2. What are the experimental studies’ retention rates, and what is the extent of users’ engagement and satisfaction with these interventions?
3. What are users’ perspectives on the use of social features in mHealth interventions to promote PA?

## Results

**Fig. 1:**
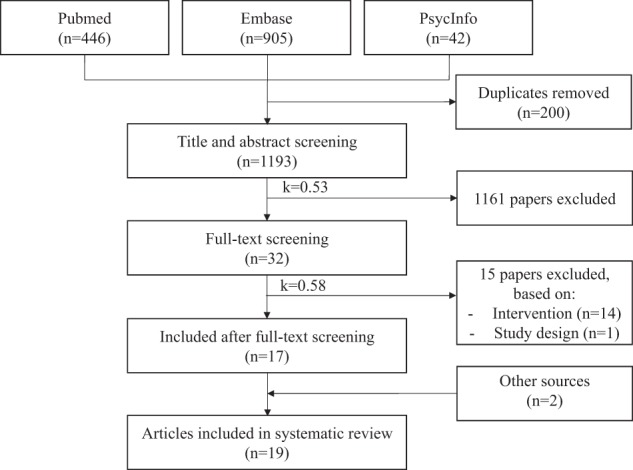
Flow diagram of included studies in which 19 studies were identified from 1393 articles in the initial database search (January 2018). Search updates were conducted until April 2018. Two additional papers were identified: one from the reference list of the included studies, one from gray literature search

The database search retrieved 1393 citations (Fig. 1); 200 duplicates were removed. After title and abstract screening, 1161 articles were excluded. Full-text screening was conducted for the remaining 32 papers, and a further 15 papers were excluded (reasons for exclusion are included in Supplement 1). Two additional papers were identified: one from the reference list of the included studies, one from gray literature search, leading to the inclusion of 19 studies for final analysis. The kappa statistic was 0.53 (fair agreement) for the title and abstract screening and 0.58 (fair agreement) for the full-text screening, before consensus agreement was reached.^19^

### Description of included studies

The final 19 studies included four RCTs,^20–23^ 10 quasi-experimental studies^24–33^ and five non-experimental studies (i.e., surveys and interviews).^36–38,39,40^ Tables 1 and 2 present a detailed characterization of the included studies. Nearly half of the studies were from the US.^21–25,29,30,32,39^ Most studies targeted healthy individuals,^20,22,23,25,27,29,31–33,36,37,39,40^ and five studies targeted specific conditions, such as chronic obstructive pulmonary disease,^38^ attention deficit hyperactivity disorder,^24^ prostate cancer,^30^ childhood cancer survivors,^21^ and stroke survivors.^26^ Publication year ranged from 2012 to 2017. Study duration in experimental studies ranged from 1 week to 6 months. Participants were diverse in age; five studies involved adolescents and young adults.^20,21,24,27,29^ Twelve studies reported no conflict of interest^20–27,31,32,38,40^ and seven studies did not include a conflict of interest statement^28–30,33,36,37,39^ (Supplement 2).

**Table 1:** Characteristics of included experimental studies

First author, year, location	Study type	Study duration	Participants *N* (I; C); *N* women; other characteristics	Intervention/study arms description	Description of social features and associated BCTs	Outcomes^a^ (*denotes significant results)	Theories and models of behavior change^b^	Retention rates I; C *N* (%)	Incentives for study compliance
Ashton, 2017, Australia^20^	RCT	3 Months	50 (26; 24); 0; Young men	2 arms I: Website + Jawbone wearable tracker + app + Facebook group + face-to-face sessions + healthy lifestyle materials C: no intervention	Facebook group *Social support*	• Steps/day • Self-reported MVPA^c^ • Feasibility	Social cognitive theory, Self- determination theory	24 (92.3%) 23 (95.8%)	Control participants received incentives for returning to the follow-up session (e.g., $10 voucher to cover travel expenses)
Mendoza, 2017, US^21^	RCT + interviews	2.5 Months	59 (29; 30); 35; childhood cancer survivors	2 arms I: Fitbit Flex tracker + Fitbit app + Facebook group + SMS C: no intervention	Facebook group *Social support*	• MVPA • Sedentary time • Motivation for PA^d^ • Enjoyment of PA^e^ • Engagement • Acceptability	Self-determination theory	29 (100%) 30 (100%)	Gift cards of “modest value” were provided to participants for completing the assessments
King, 2016, US^22^	RCT	2 Months	95 (I: 22 for analytic app, 24 for affect app, 22 for social app; C:27); 67; Inactive older adults	4 arms I: *Analytic app, Affect app, Social app* C: diet-tracker app	Social app *Social support Social comparison*	• MVPA* • Sedentary time* • EMA of brisk walking and sedentary time	*Analytic app:* Social Cognitive Theory, *Affect app:* Operant conditioning principle + Gamification*, Social app:* Social influence	Analytic app: 21 (95.5%), Affect app: 22 (91.7%), Social app: 22 (100%) Control: 24 (88.9%)	“Participants received a $20 gift card for participating”
Greene, 2012, US^23^	RCT	6 Months	513 (265; 248); NR	2 arms I: iWell OSN + wireless accelerometer + wireless scale; C: printed educational materials	iWell OSN *Social support Social comparison*	• Leisure time walking* • All physical activity • Engagement	Social network	180 (68%) 169 (68%)	Participants were compensated with a cookbook at their 3-month follow-up and a $25 Amazon.com gift card at the 6-month follow-up
Muntaner-Mas, 2017, Spain^28^	Quasi-experimental	2.5 Months	48 (I: 20 for training group, 15 for mobile group; C: 13); NR; Older adults	3 arms:I: *Training group*: in-person exercise program, *Mobile group*: WhatsApp-delivered exercise program + Chat group; C: no intervention	Mobile group: WhatsApp *Social support*	• Self-reported PA levels^f^ • Balance test • Aerobic capacity	Social network	Training group: 16 (80%); Mobile group: 7 (46.7%); Control: 9 (69.2%)	None
Schoenfelder, 2017, US^24^	Quasi-experimental + Interviews	1 Month	11 (n/a); 6; Adolescents with ADHD	1 arm: Fitbit Flex tracker + Fitbit app + Facebook group + daily text messages	Facebook group *Social support*	• Step counts* • Engagement • Acceptability^g^	NR	NR	Participants received incentives of $5/week for each online survey completed (2 per week) and $20 for the post-study interview – totaling up to $60 for adolescent and $20 for parents
Chung, 2016, US^25^	Quasi-experimental	2 Months	12 (n/a); NR; BMI = 22 – 35 kg/m^2^	1 arm: Fitbit Zip tracker + Fitbit app + Twitter	Twitter *Social support* Fitbit app *Social comparison*	• Step counts • Duration and intensity of activity • Satisfaction • Engagement	Gamification	NR	None
Paul, 2016, UK^26^	Quasi-experimental	1.5 Months	23; 12; Stroke survivors	2 arms I: Starfish app; C: no intervention	Starfish mobile app *Social comparison*	• Step counts • Sedentary time, upright time and walking time • Gait speed^h^	Behavior change techniques	NR	Participants were given compensation for travel expenses for assessment visits
Rosenberg, 2016, US^30^	Quasi-experimental + Interviews	1 week	31; 0; Prostate cancer patients	1 arm: Fitbit Zip tracker	Wearable activity trackers, i.e., Fitbit Zip *Social support*	• Acceptability	NR	26 (83.9%)	Participants kept their Fitbit and were paid $10 for completing the study
Middelweerd, 2015, Netherlands^27^	Quasi-experimental + Focus group	3 weeks	30 (n/a); 20; Dutch university students	1 arm: Nexercise app	Nexercise app *Social support Social comparison*	• Preferences, attitudes • Acceptability	NR	30 (100%)	The incentive for completing the focus groups was either an arm holder for a smartphone or voucher for free entrance to the university sports center
Pumper, 2015, US^29^	Quasi-experimental + Interviews	1 month	30 (n/a); 18; Adolescents	2 arms Group 1: Facebook group + Fitbit Flex tracker (*n* = 17) Group 2: Fitbit Flex tracker (*n* = 13)	Facebook group *Social support*	• Acceptability	NR	NR	NR
Kernot, 2014, Australia^31^	Quasi-experimental	1 month	29; 29; Women with young children	1 arm: Facebook group + pedometer	Facebook group *Social support*	• Self-reported walking*, MVPA^i^ • Feasibility • Usability • Engagement	Theory of planned behavior, Fun theory	25 (86.2%)	NR
Al Ayubi, 2014, US^32^	Quasi-experimental + Interviews	1 month	14 (n/a); NR; BMI = 18.5–43 kg/m^2^	1 arm: Persuasive Social Network for Physical Activity (PersonA) mobile app 1st week: PersonA 2nd–4th week: PersonA + social menu	PersonA mobile app *Social support Social comparison*	• Step count and distance • Usability, usefulness, feasibility, willingness to use • Accuracy	10 theories^j^	13 (92.9%)	“Participants were compensated $50 for participating”
Khalil, 2013, United Arab Emirates^33^	Quasi-experimental + Survey	2 weeks	8; 8; Pre-existing social connections	1 arm 1st week: Step up app 2nd week: Step up app + social component	Step up app *Social comparison*	• Step count • Acceptability • Satisfaction	Theory of reasoned action	8 (100%)	NR

*I* intervention, *C* control, *BCTs* behavior change techniques, *RCT* randomized control trial, *app* application, *MVPA* moderate to vigorous physical activity, *SMS* short message service, *PA* physical activity, *EMA* ecological momentary assessment, *NR* not reported, *OSN* online social network, *n/a* not applicable, *ADHD* attention deficit hyperactivity disorder, *BMI* body mass index (kg/m^2^)

^a^Outcomes reported include PA-related outcomes (e.g., steps, cognitive or psychological outcomes such as intention to exercise), engagement, acceptability, and satisfaction with the intervention. For other outcomes, see Supplement 4. ^b^As reported by the authors in the papers. Measured by: ^c^Godin Leisure-Time Exercise Questionnaire, ^d^Behavioral Regulation in Exercise Questionnaire-2, ^e^Physical Activity Enjoyment Scale, ^f^International Physical Activity Questionnaire [IPAQ]; ^g^Client Satisfaction Questionnaire [CSQ-8], ^h^Ten-Meter Walking Test (10MWT), Active Australia Survey. ^i^pre-intervention survey was developed by the authors; no validation study was published); ^j^10 theories: The Health Belief Model, the theory of reasoned action/theory of planned behavior, the Elaboration Likelihood Model, the social cognitive theory, the social support and health link theory, the uses and gratifications theory, the common bond and common identity theory, the Technology Acceptance Model, the Unified Theory of Acceptance and Use of Technology, and the Fogg Behavioral Model

**Table 2:** Characteristics of non-experimental studies

First author, year, location	Methods	Participants *N*^a^; *N* women; other characteristics	Aims	Description of mHealth technology^b^	Theories and model of behavior change mentioned^c^	Main findings
Maher, 2017, Australia^40^	Survey	237; 168; Former (*n* = 37) and current (*n* = 200) wearable tracker users	Explore users’ experience of activity trackers, including usage patterns, sharing of data to social media, perceived behavior change, and technical issues	Wearable PA trackers	NR	65% of participants said they did not use social features and 77% did not share their activity data on a social media platform. The prime motivation for using social features was reportedly “to compete with friends”
Zhu, 2017, US^39^	Survey	238; 67; Wearable trackers users	Explore the association between social competing & sharing, and intention to exercise	Wearable PA trackers	Theory of planned behavior	Social sharing and competing can directly influence attitudes towards exercise, subjective norms, and perceived behavioral control, which in turn influence intention to exercise
Stragier, 2016, Belgium^36^	Survey	394; 43; Strava (a fitness OSN) users	Test whether users’ self-regulatory motives, social motives, or enjoyment motives for fitness OSN use will predict perceived usefulness, and habitual use	Fitness OSN i.e., Strava *Social support Social comparison*	Self-determination theory	Self-regulatory motives both directly and indirectly predicted habitual use. Social motives directly predicted habitual use, while enjoyment indirectly predicted habitual use. The study also found that for new users, self-regulatory motives are the main drivers of using Strava; for experienced users, social motives and enjoyment are the main drivers
Fritz, 2014, Switzerland^37^	Interviews	30; 16; Wearable tracker users for at least 3 months	Explore factors that influence long-term use of wearable activity trackers.	Wearable PA trackers	NR	Some participants used the social features of the system but struggled to find the right community to share data with. Most users expressed the desire to share data with someone who had similar goals or interests, rather than existing social connections
Bartlett, 2017, UK^38^	Convergent mixed methods: Interviews + Survey	Interviews 28; 16; People with COPD, carers & HCPs Survey: 87; 59; People with COPD	Develop 3 prototypes of mobile apps (i.e., virtual coach system, music and maps system, online community system) and test how acceptable and persuasive each prototype is in increasing PA amongst people with COPD	Online community app *Social support Social comparison*	Persuasive System Design • Dialogue support (virtual coach) • Primary task support (music and maps) • Social support (online community)	Interviews: Opinions on social features varied between users. Some participants liked social features because of the competitiveness and communication with others who had similar experiences, while others viewed competition as unhealthy. HCPs stated that online community would be best for immobile people, but the approach would only work if the users chose it themselves. Survey: The virtual coach system was rated as most persuasive, while the online community system was rated as least persuasive. The most useful feature was instruction on how to perform behavior; while the least useful features were prompts/cues, non-specific reward and social comparison

*BCTs* behavior change techniques, *PA* physical activity, *NR* not reported, *OSN* online social network, *COPD* chronic obstructive pulmonary disease, *HCPs* health care providers

All surveys were developed by the authors; no validation studies were published. ^a^Total number of participants, ^b^behavior change techniques were classified where applicable; ^c^as reported by the authors

### mHealth technologies

Mobile apps were the most utilized technology. In experimental studies, mobile apps were used either in isolation,^22,26,28,32,33,27^ or as part of a more complex intervention with other components (e.g., wearable PA trackers).^21,23–25,29,20,31^ In two non-experimental studies, mobile apps were examined in isolation.^36,38^ Authors of seven studies developed their own apps,^22,23,26,27,32,33,38^ while the rest used the Fitbit app.^21,24,25^

Five experimental studies used wearable activity trackers as part of a multi-component intervention.^20,21,24,25,29^ Fitbit devices, such as the Fitbit Flex and Zip, were the most mentioned wearable PA trackers.^21,24,25,29,30^ Additionally, three non-experimental studies examined the use of wearable PA trackers.^37,39,40^

### Social features

In the included studies, social features were often delivered via OSNs. Specifically, four studies used Facebook,^21,24,29,31^ one used Twitter,^25^ one used WhatsApp,^28^ and one used a health-specific OSN (i.e., iWell).^23^ One study examined a fitness OSN—Strava.^36^ Social features were primarily used to deliver social support^20–22,24,25,27–32,38^ and provide social comparison.^22,25–27,32,33,38,23^ Interestingly, OSNs were also frequently used to deliver non-specific rewards (e.g., badges for PA achievements) if there has been progress in PA performance.^24,26,27,29,31^

In two experimental studies, participants mentioned that other users did not actively make use of the social features in OSNs (e.g., several users viewed posts but did not comment) and that they would like to see more engagement and contribution from others in Facebook groups.^21,29^ Other social media platforms (e.g., Snapchat, Instagram) were suggested by some younger participants as a replacement for Facebook, because they were not frequent users of the latter.^21,24^

Users’ perspectives on social features were mixed. Participants in several studies reportedly felt more motivated from social support and social comparison because they perceived a sense of membership and belonging in the group^29,32^ or because they liked the competition aspects.^27,29,33,38–40^ Meanwhile, some users said that they did not like social comparison for many reasons: (1) they were only interested in their own progress,^27,32^ (2) they thought competition might promote an unhealthy desire to win and have detrimental effects on the users’ emotions if they lose,^38^ (3) they were concerned about privacy issues.^37^ Chatroom features in mobile apps were seen as redundant in one study because the users already had other preferred communication platforms.^27^ However, they were deemed important by other participants, as they liked to have a direct way to message their friends from the app.^33^

### Behavior change techniques (BCTs) and theories

Our review found that overall, 20 of 93 possible BCTs were observed in the interventions. All interventions incorporated between 2^33^ and 14 BCTs,^20^ with a median of five BCTs per intervention. In experimental studies, self-monitoring of PA behavior was the most popular BCT, facilitated via wearable PA trackers.^20,21,23–27,29–33^ Social support was delivered in all interventions, except for two.^26,33^ Goal setting was used in six interventions.^20,24,26,30–32^ Intervention components other than the mobile technology (e.g., emails) were also used to review PA goals with participants, based on previous performance.^20,21,24^ Three experimental studies used interviews to examine which features were preferable from participants’ perspectives. The findings included goal setting, reward for progress in performing PA^24,27^ and personalized feedback.^27,30^ A complete classification of BCTs is provided in Supplement 3 (experimental studies) and Supplement 4 (non-experimental studies).

The theory of reasoned action/planned behavior was the most mentioned in the included studies,^31–33,39^ followed by self-determination theory.^20,21,36^ Social networks were mentioned twice.^20,28^ Most studies used solely one behavior change theory to inform the intervention design.^20,22,23,25–28,31–33,38^ Two non-experimental studies used behavior change theories to analyze the results.^36,39^

### Usage and acceptability

The lowest retention rate in experimental studies was 46.7% over 2.5 months.^28^ Other studies had retention rates between 68% (6-month period) and 100% (2-week period). Four studies did not report retention rates.^24–26,29^ In order to encourage participants to comply with study procedures, six studies provided incentives ranging from $10 to $25;^20–23,26,27^ three studies reported incentives of more than $50 (Table 1).^24,30,32^ Two studies did not provide any incentives,^25,28^ and three studies did not report whether they provided any incentives to participants.^29,31,33^ Chung et al. did not provide incentives for study compliance, but provided material incentives and rewards as BCTs to encourage PA behavior (i.e., complete a step challenge to get a water bottle).^25^

Measures of engagement with intervention components differed between studies, including OSN usage (e.g., liking a post on a Facebook group, sharing PA data),^21,23,24,31^ and duration of use of wearable PA trackers.^23–25,31^ Two studies found that the Fitbit tracker was worn for at least 70% of the time.^24,25^ Interestingly, Chung et al. noted that overweight participants tended to wear the Fitbit tracker 99% of the time, while normal weight participants only wore it 73% of the time (*p*-value not reported).^25^

Two non-experimental studies examined factors that influence long-term use of mHealth interventions. One study compared novice and experienced users of Strava and found that social support and social comparison were the main drivers of long-term use of the application.^36^ Another study interviewed long-term users of wearable PA trackers, and found that goal setting, reward systems, and self-monitoring were the major drivers for engagement and use.^37^ One study reported technical issues as a perceived barrier to long-term usage.^30^

User acceptability was examined in four experimental studies^21,24,27,33^ and in one non-experimental study.^38^ Even though all studies reported high levels of acceptability, only one study used a validated questionnaire;^24^ the others used interviews or surveys designed by the authors.

### Study outcomes and meta-analysis

In most studies, PA outcomes were objectively measured by a wearable tracker/pedometer^21,23–25,29–31^ or smartphone built-in accelerometers.^22,26,27,32,33^ PA outcomes were self-reported in two studies using validated questionnaires.^28,31^ One study used a pedometer to objectively measure steps per day, and used a validated questionnaire to measure self-reported moderate-to-vigorous physical activity.^20^ Six studies reported physiological outcomes (e.g., weight, Body Mass Index, blood pressure) other than PA levels (Supplement 3); one study reported cognitive and psychological outcomes (e.g., motivation for PA, enjoyment of PA).^21^

Amongst quasi-experimental studies, four reported significant increase in PA;^24–26,31^ one reported non-significant increase.^28^ Two studies also reported an increase in PA, but it was not reported if the change was statistically significant.^32,33^

**Fig. 2:**
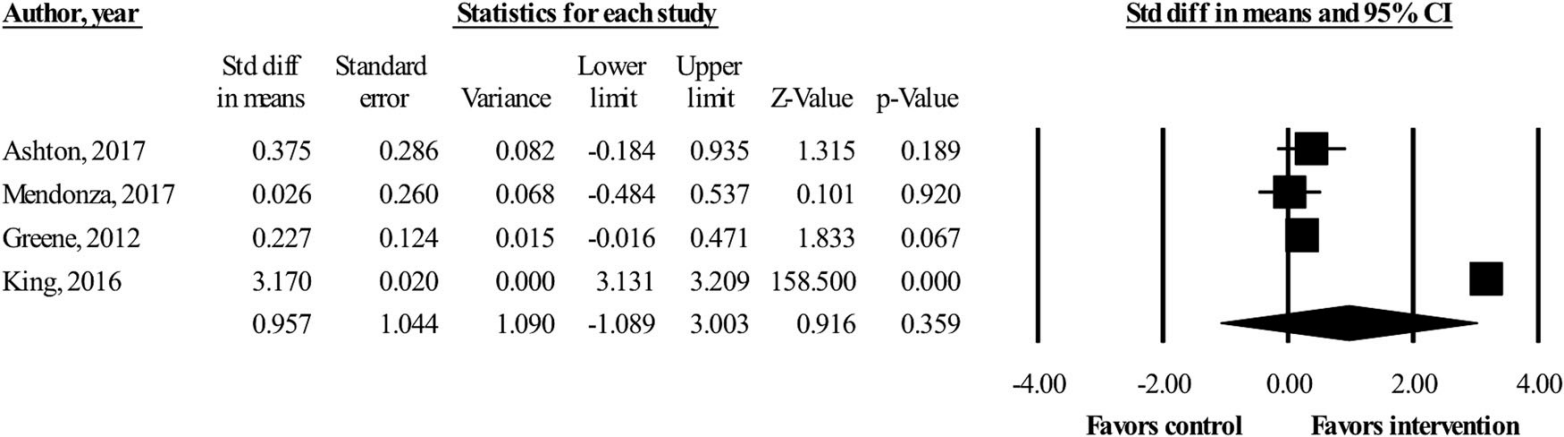
Forest plot of effect sizes and 95% confidence intervals (CI) representing the effect of mobile health interventions with social features on physical activity outcomes (random effects model)

We included four RCTs in the meta-analysis, all with continuous outcomes.^20–23^ There was no statistically significant effect of mHealth interventions with social features on PA outcomes [standardized difference in means = 0.957 (95% confidence interval −1.09 to 3.00)] (Fig. 2). Heterogeneity was high (*I*^2^ 99.6%).

### Risk of bias assessment

Out of four included RCTs, two studies were deemed as having the lowest risk of bias according to Cochrane’s tool (low risk of bias in five out of six categories,^20^ and four out of six categories^22^) (Supplement 5). All studies had a low risk of bias for random sequence allocation, and a high risk of bias for blinding of participants and personnel. Two studies lacked sufficient information for risk assessment in allocation concealment,^21,23^ and blinding of outcome assessment.^21^ Even though all four studies mentioned trial registration, one failed to provide the registration identification number,^23^ and another had very limited information on the registration,^21^ which made it difficult to assess “selective reporting”. Included studies other than RCTs had a higher risk of bias; detailed assessment was not possible due to the quality of reporting.

## Discussion

### Main findings

The integration of social features in mHealth for PA promotion appears to be in an early stage of development due to the recent timing of publication of included studies (all published after 2010), and the predominance of quasi-experimental studies. Social features were often delivered via OSNs and used to provide social support or social comparison. From users’ perspectives, preferences and use of social features were mixed: some users felt more motivated because of social support and competition aspects, while others expressed concerns about engaging in social comparison.

### Comparisons with existing literature

Our systematic review focuses on the integration of social features in mHealth technology to promote PA. Several systematic reviews examined the use of mHealth technology to promote PA;^12–18^ however, none has focused on social features.

Two recent systematic reviews have looked at the effectiveness of OSNs on health behavior change,^11,35^ and found modest effects on health outcomes. These two systematic reviews differ from our study in several ways. Firstly, this study focuses solely on PA, while other studies looked at a range of health behaviors. Secondly, instead of examining OSNs (which can be web-based or delivered as a software application), we examined social features providing BCTs (e.g., social support, social comparison) in mHealth. Thirdly, rather than including only experimental studies, our review also included non-experimental studies such as surveys and interviews to capture users’ perspectives on the use of social features. Notably, even potentially efficacious interventions can fail to have an impact if users do not adopt the technology or use it over a long period of time. Thus, it is important to understand users’ perspectives on engagement with mHealth to inform intervention development and implementation.

### The use of social features and BCTs in mHealth

Our study found that social features were most often used to deliver social support and social comparison. We also observed that self-monitoring of behavior was the most commonly used BCT in the included studies, which is in line with findings from previous literature.^12,13,15^ Self-monitoring of behavior can be seen as an important starting point to provide other BCTs,^13^ such as social comparison, or provision of feedback. A previous meta-analysis has shown that PA interventions that included self-monitoring and at least another self-regulatory technique (e.g., goal setting, feedback, on behavior) were significantly more effective than other interventions.^41^ While these findings shed light into the common use of BCTs in health interventions, due to the quasi-experimental nature of most studies, it remains unclear whether specific bundles of BCTs are more effective than others. An interesting hypothesis (which remains untested) is that different BCTs might be effective in different stages of behavior change,^42^ indicating the promises of adaptive interventions, tailored to individual progress.

Additionally, from users’ perspectives, preferences for social features were mixed amongst the participants in several included studies,^27,29,32,33,37,38,40^ which could be linked to differences in individual characteristics. For example, some participants acknowledged that they liked social comparison because of their own competitive nature.^38^ In contrast, other users showed interest in self-comparison only, preferring to follow their own goals and plans, and seeing little benefit in comparing themselves with other people.^32^ This indicates that while some BCTs (e.g., self-monitoring) might be suitable for most users, others (e.g., social comparison) might be more controversial, and thus, users’ preferences and characteristics should be taken into account when delivering an intervention, rather than adopting a one-size-fits-all approach.

### User engagement and retention

Retention rates of included studies were generally high. Specifically, four studies reported a 100% retention rate,^21,22,27,33^ and four studies reported at least 80%.^20,30–32^ The only exception is the Muntaner-Mas study with a retention rate of <50%.^28^ The use of social features in the Muntaner-Mas was considerably limited (i.e., only the chat function of WhatsApp was used), and no incentives for study completion were provided, which might explain the lower retention rate.

The high retention observed in most included studies suggests that integrating social features into mHealth interventions could potentially increase user engagement and retention, addressing the common attrition problem in health informatics studies.^43^ Other systematic reviews have reported high retention rates for behavioral informatics interventions that incorporated general OSNs (e.g., Facebook).^11,35^ A recent longitudinal study has examined a large dataset of six million users over 5 years to determine whether social networking features influence user engagement, or change behavior within the application, as well as in real life. By comparing social network users to matched control non-users, the study observed a 17% increase in user retention for social network users, with the long-lasting effect of over 1 year.^44^

Another aspect worth considering is the use of incentives and rewards. It is important to draw the distinction between incentives for study compliance (e.g., compensation of $10 for traveling to the research center) and incentives used as BCTs, targeting a particular behavior (e.g., offering a prize when a certain number of steps is achieved).^45^ In terms of incentives for study compliance, research has shown that these can influence retention rates.^46,47^ In this review, due to the multi-component nature of the included interventions and the study designs used, it is not possible to distinguish between the different impact of social features and compliance incentives on retention rates. In terms of incentives targeting behavior, several studies have demonstrated their potential effectiveness.^48–50^ However, researchers have questioned whether providing material incentives may undermine the development of intrinsic motivation and impact autonomy in decision-making^51–53^—factors which are strongly predictive of long-term exercise adherence.^54^ Questions have also been raised about the scalability and sustainability of material incentives, highlighting the need to explore sustainable incentive procedures in future research.^55^

### Strengths and limitations

There are several strengths in our study. Prior to the study commencement, we developed and registered a protocol in the PROSPERO database, which we followed systematically throughout the study. The screening form was also pre-tested and piloted before screening began. Furthermore, all the studies were independently screened by two researchers; a kappa score of 0.53 (first round) and 0.58 (second round) revealed a fair level of agreement. Lastly, BCTs were coded using a pre-tested and validated taxonomy,^45^ which provided an objective way to examine how BCTs have been used in social features and mHealth. The BCTs were coded and reviewed by two researchers who have achieved coding competence in the use of BCTTv1.

Our findings should be interpreted in light of some limitations. Firstly, through our database search, we were unable to find a complete and sound definition of social features. Instead, we developed our own definition of social features based on the literature. Secondly, we excluded papers that were not in English. Even though this was done to ensure that the authors could fully understand and make an informed decision in the screening phase, we might have missed some important papers. Thirdly, for our review, we classified BCTs according to the intervention description provided in the papers and did not infer the presence of BCTs, potentially leading to a lower overall number of BCTs found compared to other reviews.^12,13^ Fourthly, the short study duration and the incentives provided by some included studies could potentially influence the observed retention rates. Finally, the predominance of low-quality experimental studies and the heterogeneity of the RCTs reflected the emerging nature of this field, which limited our ability to draw strong conclusion regarding the intervention effectiveness on PA.

### Implications for research

Our study highlights several important implications on potential research areas and study design. Firstly, our findings suggest that self-monitoring of behavior seems to be prevalent and relevant in PA interventions. While social features appear to be important to user engagement and retention, due to the limited number of RCTs and the multi-component nature of the interventions, it was difficult to ascertain their impact on retention, or their effectiveness on PA outcomes. It is important to note that material incentives could also contribute to high retention or be used as a BCT. However, questions about the sustainability of material incentives remain, suggesting the need to explore other kinds of incentives (e.g., social, verbal encouragement or virtual prizes).^55^ Users’ mixed preferences regarding social features and BCTs suggest that a one-size-fits-all approach might be inadequate, highlighting the need to personalize interventions based on individual characteristics and preferences.

To develop and assess personalized interventions with multiple components and BCTs (e.g., incentives, social features), future studies should consider using factorial and adaptive study designs. The Multiphase Optimization Strategy and the Sequential Multiple Assignment Randomized Trial may be particularly useful to determine which intervention components or combinations are most effective, what is the optimal sequence for delivering these components, and which tailoring variables should be used.^56^ Furthermore, authors are urged to follow the Consolidated Standards of Reporting Trials for electronic and mobile health applications and online telehealth (CONSORT-EHEALTH),^57^ and the Transparent Reporting of Evaluations with Nonrandomized Designs (TREND) statement when reporting their findings, in order to increase evidence quality and facilitate future reviews and meta-analyses.^58^

## Methods

For the purpose of this systematic review, we defined social features within mHealth PA interventions as those that enable the interaction of an individual with other people (e.g., OSNs), and/or the delivery of *social* BCTs (e.g., social support, social comparison).^45^ As the domain of mHealth is broad, we specifically focused on the use of mobile apps and wearable PA trackers.

### Search strategy

A systematic search of the literature was performed in January 2018, and updated in April 2018, using PubMed, Embase, and PsycInfo. Search strings included several terms related to mobile health and social features (a complete search strategy is provided in Supplement 6). No restrictions were placed in the search according to the year of publication. We also searched the reference lists of relevant articles and gray literature (e.g., dissertations, theses, conference proceedings). Authors were contacted when additional information about the studies was needed.

### Study selection criteria

We included any primary research studies that involved patients or healthy consumers who used or were exposed to a mobile health intervention with social features, where the primary aim was to promote PA (e.g., increase step counts, intention to exercise). As we wished to examine both intervention effectiveness and users’ perspectives on mHealth interventions with social features, we included both quantitative and qualitative studies.

Studies were excluded if they: (1) did not incorporate social features in the mHealth component of the intervention; (2) involved only short message service (SMS), web (i.e., applications that are solely web-based), telephone, telemonitoring or telemedicine, or static pedometers (i.e., not able to transmit data to a consumer interface); (3) only reported PA as a secondary outcome or did not mention PA at all; (4) were not in English.

### Screening, data extraction, and synthesis

Two investigators piloted the screening procedure and independently conducted two-phase screening: (1) title and abstract and (2) full-paper screening. Cohen’s kappa was used to measure inter-coder agreement in each screening phase. Disagreements were resolved through discussion and consensus.

One investigator extracted information from the included studies into a standardized form; another investigator examined the form for consistency. The following data were collected for each study: first author, year of publication, location, study duration, type of mHealth technology, social features, intervention components and characteristics, participants and setting information, reported outcomes, incentives for study compliance, conflicts of interest and funding sources. For each intervention component, BCTs were coded according to the BCT Taxonomy v1^45^ and reviewed by two researchers with coding competency. Decisions on coding were made based on the authors’ description of the interventions. Though there is a specific CALO-RE taxonomy on physical activity and healthy eating,^59^ we chose the BCT Taxonomy v1 as it is the most comprehensive and up-to-date classification. For randomized controlled trials (RCTs), study quality was assessed using Cochrane’s risk of bias tool.^19^

We conducted a narrative synthesis of results for all studies, and a meta-analysis for RCTs. We transformed all effect sizes to a common metric comparable across studies—the bias-corrected standardized difference in means—and classified it as positive when in favor of the intervention and negative when in favor of the control. We used a random effects model to combine the results in a more conservative way. As suggested in the literature, we did not avoid conducting a meta-analysis based on heterogeneity.^60–62^ Instead, we assessed the presence of heterogeneity using I^2^ statistics and cautioned readers in the interpretation of the results.^61,62^ Due to the small number of included RCTs, a subgroup analysis was not conducted. Comprehensive Meta-Analysis V.2.2 was used for computations.

The study protocol was registered with PROSPERO (International prospective register of systematic reviews) with number CRD42018086067. This systematic review is compliant with the Preferred Reporting Items for Systematic Reviews and Meta-Analyses (PRISMA) statement.^63^

## Conclusion

The integration of social features in mHealth interventions for PA is a new field of research that has potential to increase user engagement and physical activity. Future research should adopt innovative research designs to develop and evaluate multi-component personalized interventions for PA promotion.

## Electronic supplementary material

Supplementary information

## Data Availability

The authors declare that the data supporting the findings of this study are available within the paper and its supplementary information files.

## Electronic supplementary material

**Supplementary information** accompanies the paper on the *npj Digital Medicine* website (10.1038/s41746-018-0051-3).
