# Supplementary material for: The use of social features in mobile health interventions to promote physical activity: a systematic review
Source: NPJ Digit Med. 2018 Sep 4;1:43. doi: 10.1038/s41746-018-0051-3 (PMC6550193; doi:10.1038/s41746-018-0051-3)
Supplement: Supplementary file 1 — Supplementary information [file 41746_2018_51_MOESM1_ESM.pdf]

Supplementary Information File 1

List of articles excluded after full-text review, for not meeting inclusion criteria regarding the population, intervention, outcome or study design

Intervention:

1. Adams MA, Sallis JF, Norman GJ, *et al*. An adaptive physical activity intervention for overweight adults: A randomized controlled trial. *PLoS One* 2013;8(12): e82901
2. Albright CL, Maddock JE, Nigg CR. Increasing physical activity in postpartum multiethnic women in Hawaii: Results from a pilot study. *BMC Womens Health* 2009;9(4)
3. Brett CE, Pires-Yfantouda R. Enhancing participation in a national pedometer-based workplace intervention amongst staff at a Scottish university. *Int J Health Promot Educ* 2017;55(4):215-28
4. Cavallo DN, Tate DF, Ries AV, *et al*. A social media-based physical activity intervention: a randomized controlled trial. *Am J Prev Med* 2012;43(5):527-32 doi: 10.1016/j.amepre.2012.07.019.
5. Chapman GB, Colby H, Convery K, *et al*. Goals and social comparisons promote walking behavior. *Med Decis Making* 2016;36(4):472-8 doi: 10.1177/0272989x15592156.
6. Livingood WC, Monticalvo D, Bernhardt JM, *et al*. Engaging adolescents through participatory and qualitative research methods to develop a digital communication intervention to reduce adolescent obesity. *Health Educ Behav* 2017;44(4):570-80 doi: 10.1177/1090198116677216.
7. Monroe CM, Bassett DR, Jr., Fitzhugh EC, *et al*. Effect of adding online social support tools to an adult walking program: A pilot randomized controlled trial. *Health Promot Pract* 2017;18(1):84-92
8. Rote AE, Klos LA, Brondino MJ, *et al*. The efficacy of a walking intervention using social media to increase physical activity: A randomized trial. *J Phys Act Health* 2015;12 Suppl 1:S18-25 doi: 10.1123/jpah.2014-0279.
9. Wojcicki TR, Grigsby-Toussaint D, Hillman CH, *et al*. Promoting physical activity in low-active adolescents via Facebook: A pilot randomized controlled trial to test feasibility. *JMIR Res Protoc* 2014;3(4):e56 doi: 10.2196/resprot.3013.
10. Xian Y, Xu H, Liang L, *et al*. An initial evaluation of the impact of Pokemon GO on physical activity. *J Am Heart Assoc* 2017;6(5): e005341
11. Joseph RP, Keller C, Adams MA, *et al*. Print versus a culturally-relevant Facebook and text message delivered intervention to promote physical activity in African American women: a randomized pilot trial. *BMC Womens Health* 2015; 15:30 doi: 10.1186/s12905-015-0186-1.
12. Maher C, Ferguson M, Vandelanotte C, *et al*. A web-based, social networking physical activity intervention for insufficiently active adults delivered via Facebook app: Randomized controlled trial. *J Med Internet Res* 2015;17(7):e174 doi: 10.2196/jmir.4086.
13. McMahon SK, Lewis B, Oakes J, *et al*. Assessing the effects of interpersonal and intrapersonal behavior change strategies on physical activity in older adults: A factorial experiment. *Ann Behav Med* 2017;51(3):376-90
14. Plotnikoff RC, Wilczynska M, Cohen KE, *et al*. Integrating smartphone technology, social support and the outdoor physical environment to improve fitness among adults at risk of, or diagnosed with, Type 2 Diabetes: Findings from the 'eCoFit' randomized controlled trial. *Prev Med* 2017; 105:404-11

Study design:

15. Kernot J, Olds T, Lewis LK, *et al*. Effectiveness of a Facebook-delivered physical activity intervention for post-partum women: a randomized controlled trial protocol. *BMC Public Health* 2013; 13:518 doi: 10.1186/1471-2458-13-518.

Supplementary Information File 2

Conflict of interests and funding sources of included studies

Paper	Conflict of interest declaration	Funding
Ashton, 2017[20]	"The authors declare that they have no competing interests."	"HEYMAN was funded by a Hunter Medical Research Institute (HMRI) project grant. L.M. Ashton undertook this research as part of a requirement for the degree of PhD (Nutrition and Dietetics), The University of Newcastle, Australia. L.M. Ashton is supported by an International Postgraduate Award Scholarship and The Greaves Family Medical Research Scholarship through HMRI. CEC is supported by a National Health and Medical Research Council of Australia Senior Research Fellowship."<br>"The research was funded by a project grant from the Hunter Medical Research Institute (HMRI) (14–30). HMRI did not have any influence on the performance of the trial, analysis of the data, writing, or the publication of the results. CEC is supported by an NHMRC Senior Research Fellowship."
Mendoza, 2017[21]	"The authors declare that there is no conflict of interest."	"This study was supported by a Supportive Care Research Grant from St. Baldrick's Foundation and matching internal funding from the Adolescent and Young Adult Cancer Program of Seattle Children's Hospital. The funders had no role in the design, collection, analysis, and interpretation of data or writing/submission of this report."
King, 2016[22]	"The authors have declared that no competing interests exist."	"This work was supported by US Public Health Service grant #RC1 HL099340 from the National Heart, Lung, & Blood Institute of the National Institutes of Health (NIH) awarded to Dr. King; US Public Health Service Grant 1U54EB020405 supporting The National Center for Mobility Data Integration and Insight; and US Public Health Service grant #5T32L007034 from the National Heart, Lung, & Blood Institute. The funders had no role in study design, data collection and analysis, decision to publish, or preparation of the manuscript."
Greene, 2012[23]	"The author(s) declared no potential conflicts of interest with respect to the research, authorship, and/or publication of this article."	"The author(s) disclosed receipt of the following financial support for the research, authorship, and/or publication of this article: Financial support for conducting the clinical trial was provided by SK Telecom Americas."
Muntaner-Mas, 2015[28]	NR	NR

Schoenfelder, 2017[24]	“None”	“Funding was provided by the Center for Child Health, Behavior and Development at Seattle Children's Research Institute, 2015.”
Chung, 2016[25]	“The author(s) declared no potential conflicts of interest with respect to the research, authorship, and/or publication of this article.”	The author(s) disclosed receipt of the following financial support for the research, authorship, and/or publication of this article: Funding for this project was received from the Academic Pediatric Association’s Young Investigator Award and the NC Children’s Promise Grant. Dr Chung receives funding support from the National Center for Advancing Translational Sciences, National Institutes of Health, through grant 1KL2TR001109.”
Paul, 2016[26]	“The authors declare no conflicts of interest.”	“This work was supported by Chest Heart & Stroke Scotland [CHSS Ref: Res146].”
Rosenberg, 2016[30]	NR	NR
Middleweerd, 2015[27]	“The authors declare that they have no competing interests.”	“This research is supported by Philips and Technology Foundation STW, Nationaal Initiatief Hersenen en Cognitie NIHC under the Partnership program Healthy Lifestyle Solutions (grant no 12014).”
Pumper, 2015[29]	NR	NR
Kernot, 2014[31]	“The authors have declared that no competing interests exist.”	“The software development for the Mums Step it Up Facebook app was funded by a research development grant from the Division of Health Sciences at the University of South Australia (http://www.unisa.edu.au). JK is supported by an Australian Postgraduate Award Scholarship. CM is supported by an Australian Research Council Australian Postdoctoral Fellowship. The funders had no role in study design, data collection and analysis, decision to publish, or preparation of the manuscript.”
Al Ayubi, 2014[32]	“None declared”	“This work is partly funded by the following grants, Grant #R25 RR023274-03 from the National Center for Research Resources, National Institutes of Health, United States; Grant #1R21HD071810-01A1 from the Eunice Kennedy Shriver National Institute of Child Health and Human Development, National Institutes of Health, and Grant # SC090323 from the Department of Defense.”
Khalil, 2013[33]	NR	“We would like to thank Emirates Foundation for their support to that research project.”
Maher, 2017[40]	“The authors declare that they have no competing interests.”	“The authors have no funding to declare.”
Zhu, 2017[39]	NR	NR
Stragier, 2016[36]	NR	NR
Fritz, 2014[37]	NR	“This work was partially funded by the Natural Sciences and Engineering Research Council of Canada (NSERC).”

Bartlett, 2017[38]	"None declared"	"The work was funded by the EPSRC. The sponsors did not have a role in the preparation or publication of this manuscript. This research was supported by the NIHR Collaboration for Leadership in Applied Health Research and Care Yorkshire and Humber (NIHR CLAHRC YH). The views and opinions expressed are those of the authors and not necessarily those of the NHS, the NIHR, or the Department of Health."

Abbreviation: NR: not reported

Supplementary Information File 3

Additional information about experimental studies

First author, year	BCTs	Associated quotes	Other outcomes	Main results
Ashton, 2017[20]	Private Facebook discussion group<br>*3.1 Social support (unspecified)*<br>Website<br>*4.1 Instruction on how to perform the behavior*<br>*6.1 Demonstration of the behavior*<br>Jawbone wearable tracker +app<br>*1.1 Goal setting (behavior)*<br>*2.3 Self-monitoring of behavior*<br>One-hour weekly face-to-face sessions with researchers<br>*1.1 Problem solving*<br>*1.5 Review behavior goal(s)*<br>*2.2 Feedback on behavior*<br>*4.1 Instruction on how to perform the behavior*<br>*8.1 Behavior practice/rehearsal*<br>*8.3 Habit formation*<br>*9.1 Credible source*<br>Gymstick resistance band<br>*12.5 Adding objects to the environment*<br>TEMPlate Dinner disc<br>*4.1 Instruction on how to perform the behavior*	3.1 "facilitate social support"<br>4.1 "a 'resource library' housing relevant information and resources, including fact sheets from best practice guidelines, […] and recommended mobile applications for improving eating habits, physical activity, reducing alcohol intake or coping with stress"<br>6.1 "support videos (e.g. short cooking videos and demonstration of Gymstick™ exercises)"<br>1.1 "goal setting"<br>2.3 "self-monitoring of key health behaviors"<br>1.4 "a mixture of practical (e.g., mindfulness-based stress reduction) and theoretical (e.g., problem solving strategies to address key issues apparent in young men, i.e., lack of money) components"<br>1.5, 2.2 "personalized feedback from a food and nutrient report (see below), and from the Jawbone physical activity data. From this, personal tailored goals were set."<br>4.1 "healthy eating education (e.g., meal planning and meal ideas for quick, cheap and healthy meals)"<br>8.1 "practical exercise activities focusing on aerobic (e.g., team based recreational games) and strength exercises (e.g., High Intensity Interval Training)"<br>8.3 "Group based sessions took place on Thursday evenings (18:00–19:00 pm)"<br>9.1. "Sessions were delivered by two male researchers from the same age demographic (one was a qualified P.E. teacher, undertaking a PhD in Education and the other was a PhD candidate in Nutrition and Dietetics)"<br>12.5 "A Gymstick™ resistance band, for home-based strength training with linked routines available on the website"<br>4.1 "guide main meal portion size for main meal components"	• Weight<br>• Fat mass & skeletal muscle mass<br>• BMI<br>• Cholesterol (mmol/L)<br>• Blood pressure (mmHg)<br>• Resting heart rate<br>• Diet quality (Australian Eating Survey)<br>• Alcohol consumption (Alcohol Use Disorders Identification Test–consumption scale)<br>• Subjective well-being (Satisfaction with Life Scale)<br>• Self-reported measures of mental health and well-being (Kessler psychological distress scale, Depression Anxiety Stress Scale, Mental Health Continuum-Short Form, Quality of Life, Enjoyment & Satisfaction Questionnaire)	Participants reported frequent usage levels for most program components, other than Facebook discussion group and some of the materials on diet. They also gave a score of 3 – 4.6/5 for the program component acceptability.<br>There was no significant change in steps/day, or total wellbeing score. Significant effects were found for daily vegetable servings, energy-dense, nutrient-poor food, MVPA< weight, BMI, fat mass, waist circumference and cholesterol.

Mendoza, 2017[21]	Facebook group<br>*3.1 Social support (unspecified)*<br>*10.3 Non-specific reward*<br>Fitbit Flex tracker + app<br>*2.2 Feedback on behavior*<br>*2.3 Self-monitoring of behavior*<br>SMS from researchers<br>*1.7 Review behavior goal(s)*<br>*7.1 Prompts/cues*	3.1 "a forum for participants to encourage and discuss their experiences using the Fitbit and their PA goals"<br>10.3 "The Facebook group was moderated by research staff, who provided intervention participants with badges for PA and participation achievements every week"<br>2.2 "displays progress towards a personalized goal"<br>2.3 "provide estimates of steps, very active minutes, energy expended (calorie burned) and distance travelled"<br>1.7 "Help set a daily step goal based on mean step counts for week 1", "Gradually increase their step count goals to meet or maintain population recommendations for adolescents"<br>7.1 "sent affective text messages for PA every other day to encourage and remind intervention participants about their PA goals"	• Health-related quality of life (Pediatric Quality of Life Inventory 4.0 Generic Core and Cancer Module Scales)	There were no significant adjusted group differences for change in MVPA or sedentary time between intervention and control group. For PedsQL, social functioning scale of was the only one experienced significant adjusted difference i.e. intervention groups decreased from 86.1 to 83.9; while control group increased from 78.8 to 84.7. Intervention group also experienced increased introjected motivation (internalizing external pressure that leads to the desired behavior).<br>92.3% participants saw at least one post, 65.4% commented on at least one post. Liking posts was the lowest type of engagement (50%).<br>Qualitative data revealed that participants found the Fitbit Flex and Facebook acceptable and helpful. Participants expressed the desire for more activity on the Facebook group; while many recommended the use of Snapchat and Instagram instead.
King, 2016[22]	Social app<br>*3.1 Social support (unspecified)*<br>*6.2 Social comparison*<br>Analytic app<br>*1.1 Goal setting (behavior)*<br>*2.2 Feedback on behavior*<br>*4.1 Instruction on how to perform a behavior*<br>Affect app<br>*2.2 Feedback on behavior*<br>*10.3 Non-specific reward*	3.1 "social support", "group-based collaboration", "online message board"<br>6.2 "just-in-time social normative feedback", "group-based […] competition"<br>1.1, 2.2 "personalized and quantified goal-setting and behavioral feedback"<br>4.1 "informational tips or advice for behavior change"<br>2.2 "The bird avatar, which was viewable on the phone's glance-able display throughout the day, changed position, posture, and movement depending on how active or inactive the user was up to that time point."<br>10.3 "rewards" (e.g., the bird avatar would unexpectedly appear in far-away cities) as a function of increased physical activity levels"	n/a	The social app users showed significantly greater increase in MVPA compared to the other arms, and significantly lowered sedentary behavior. For the Ecological Momentary Assessment of brisk walking variable, there were no significant effect for time or significant differences between study arms. For reported sitting time, the social and affect apps both reported significantly less sitting time than the Analytic app or control group.
Greene, 2012[23]	iWell OSN<br>*3.1 Social support(unspecified)*<br>*6.2 Social comparison*<br>Wireless accelerometer + wireless scale<br>*2.3 Self-monitoring of behavior*<br>*2.4 Self-monitoring of the outcome of behavior*	3.1 "participants could connect ("friend") others in the network, send individual messages to their friends, make public postings, view their contact's postings…"; 6.2 "view their physical activity or "steps," view their weight", "compete against others in the network on the number of "steps" walked or run"<br>2.3 "given an accelerometer that allowed them to capture their physical activity or steps"	• Weight<br>• Triglycerides<br>• Low-density & high-density lipoprotein	The intervention group was found to have significantly increased their weekly leisure walking from 129 to 341 minutes (164% increase) over six months, while the control group increased by 47%.<br>The intervention group also lost more weight than the control group (5.2 vs 1.6 pounds).

	Paper-based materials<br>*1.1 Goal setting (behavior)*<br>*4.1 Instruction on how to perform the behavior*<br>*5.1 Information about health consequences*	2.4 "[…] a wireless weight scale for uploading weight data"<br>1.1, 4.1, 5.1 "All participants received printed lifestyle guidelines on diet and exercise (...); sample daily meal plan with recommended serving sizes, a handout about recommended daily levels of exercise, and a number of articles about the benefits of exercise and healthy eating."		There was no significant difference between the two groups in changes in low-density and high-density lipoprotein. Amongst the intervention group, only the number of messages sent by participants was positively related to increased leisure walking ($p<0.05$), and negatively related to weight change ($p<0.01$).
Muntaner-Mas, 2015[28]	Mobile group: WhatsApp<br>*3.1 Social support (unspecified)*<br>*4.1 Instruction on how to perform the behavior*<br>*7.1 Prompts/cues*<br>Training group: In-person training sessions<br>*4.1 Instruction on how to perform the behavior*<br>*8.1 Behavior practice/rehearsal*	3.1 "a mobile phone app based on a social network", "all participants were added to a chat group"<br>4.1 "received 2 videos […] per week for 10 weeks", "the content of them were the exercise sessions", "the videos were attached to the group chat", "a member of the research group carrying out the prescribed exercises"<br>7.1 "the administrator sent out 2 messages in the group chat per week, which reinforced messages from the videos, and encouraged participants to perform physical exercise"<br>4.1 "training sessions on the sports ground (Mondays and Wednesdays)"<br>8.1 "involved participant exercising", "repetitions"	• Blood pressure<br>• Waist circumference<br>• Weight-to-height ratio<br>• BMI<br>• Fat-mass and fat-free mass index<br>• Handgrip strength	The Mobile group increased handgrip strength, aerobic capacity and decreased systolic blood pressure and heart rate after exercise though there were no significant differences respect to Control group. The Training group decreased significantly blood pressure and heart rate after exercise, respect to Control group. Diastolic blood pressure decreased significantly more in Training group than Mobile group.<br>There were no other significant differences between the intervention and control group, or between the Training and Mobile group.
Schoenfelder, 2017[24]	Facebook group<br>*3.1 Social support (unspecified)*<br>*10.3 Non-specific reward*<br>Fitbit Flex + app<br>*1.1 Goal setting (behavior)*<br>*2.2 Feedback on behavior*<br>*2.3 Self-monitoring of behavior*<br>Emails from researchers<br>*1.5 Review behavior goals*	3.1 "interact with other participants", "a study staff posted to the group, interacted with participants, and monitored posts daily" "participants were encouraged, but not required, to post in the group, encourage their fellow participants, and share their Fitbit data on Fb."<br>10.3 In the Facebook group, "participants earned digital badges for meeting weekly activity goals, as well as for social interactions<br>(e.g., liking other's posts) or making improvement towards goals"<br>2.2 "feedback toward personalized goal attainment"<br>2.3 "collect data through its built-in accelerometer to provide proxy estimates of PA including steps, energy expended, and distant travelled", "provide graphs of the data"<br>1.5 "an individualized goal based on their average week 1 steps plus 1% steps weekly"	• ADHD symptoms (Vanderbilt ADHD Diagnostic Parent Rating Scale)<br>• Mood valence (10-item Positive and Negative Affect Schedule for Children)	There was a significant increase in step counts (3218 in total, 95% CI: 931 to 5291, 107 steps/day).<br>There was also a significant decrease in teen and parent-reported Inattentive and Hyperactive/Impulsive symptoms (-0.4 to -0.8). There was no significant change in mood valence. Total score for acceptability was 1.4 for both adolescents and parents (1=definitely, 4=not at all). In qualitative interviews, participants reportedly said that they had positive experiences with the study, and increased awareness of their PA level and ADHD symptoms. The most common suggestions were increasing reminders, adding additional challenges/goals, and using other social media sites i.e. Instagram.
Chung, 2016[25]	Twitter<br>*3.1 Social support (unspecified)*<br>*2.2 Feedback on behavior*<br>Fitbit app<br>*6.2 Social comparison*	3.1 "post questions to the study team or to their Twitter group", "received photo-based Twitter messages that were pictures of healthy food options, infographics, and website links related to healthy lifestyle tips"	• Weight<br>• Body fat percentage<br>• Self-reported food intake and lifestyle changes	The participants were categorized as overweight/obese (BMI 25 – 34.9 kg/m^2) or healthy weight (BMI 22.5 – 24.9 kg/m^2). Overweight participants (OW) had 11,222 daily steps on average vs 11,686 steps for

	Fitbit Zip tracker<br>*2.3 Self-monitoring of behavior*<br>Study team<br>*10.1 Material incentive (behavior)*<br>*10.2 Material reward (behavior)*	2.2 “Personalized step challenges based on their physical activity patterns during the previous month”<br>6.2 “Fitbit accounts were set up to auto-tweet daily steps and distance travelled to the assigned private Twitter group so that individuals could see how others were doing, which was the basis of some of the competitions”, “individual vs group challenges”<br>2.3 “measure steps, physical activity intensity and duration, and caloric expenditure”, “displays the number of steps, miles travelled, and caloric expenditure on the small screen within the device so that users can view their data at any time”<br>10.1, 10.2 “Throughout the study period, the study team created individual vs. group challenges, including personalized step challenges, most steps/day or per week within groups, and so on, to determine whether participants could be incentivized to make behavioral changes using principles of gamification. We provided prizes that were $10 or less but provided regular challenges to facilitate ongoing engagement (i.e. water bottles, weights, etc.). We also periodically challenged participants to beat their own personal average steps/day.”		healthy weight (HW). Overall, there was an increase in PA during the challenges.<br>92% participants self-reported increased fruit intake; OW increased by 2.1 servings vs 1.8 servings (HW). 58% self-reported increased vegetable intake (2.5 servings for OW, 0.5 servings for HW). OW lost one to five pounds, and 3.9% to 10.6% body fat vs 0.2 to 7 pounds, and 0.5% to 13.5% for HW.<br>100% participants reported being very likely/likely to recommend the intervention to others. Compliance with daily Fitbit wear was 99% of all days for OW and 73% for HW.
Paul, 2016[26]	Starfish mobile app<br>*1.1 Goal setting (behavior)*<br>*1.5 Review behavior goals*<br>*2.3 Self-monitoring of behavior*<br>*6.2 Social comparison*<br>*10.3 Non-specific reward*	1.1 “individualized step goals were set for each person”<br>1.5 “In week one, the daily step count target was the mean number of steps per day recorded on the phone during the baseline period (see below) plus 10%. At the end of each week, if individuals achieved their step count target on five of seven days, their target for the following week was increased by 5%. This update was indicated to the user by an exclamation mark attached to their fish. If the target was not reached, it remained unchanged for the following week.”<br>2.3 “When the participant is active their fish swims and blows bubbles which they, and other participants, can see”<br>6.2 “When the participant is active their fish swims and blows bubbles which they, and other participants, can see”<br>10.3 “Individual and group “rewards” for achieving goals were provided. As the participant reached their target number of steps, their fish’s fins and tail grew. If all four members reached their step count target on at least five days of the week then the group was rewarded by another sea	• Weight<br>• BMI<br>• Resting heart rate<br>• Blood pressure<br>• Impact of fatigue (Fatigue Severity Scale)<br>• Complex activities of daily living necessary for functioning in community settings (Instrumental Activities of Daily Living Scale)<br>• Quality of life (Stroke Specific Quality of Life Scale)<br>• Subjective well-being (Psychological	The mean number of steps/day increased by 39.3% (1633 steps/day) in intervention group; while it decreased by 20.2% (747 steps/day) for the control group. Walking time also increased by 20 mins/day for the intervention group, and reduced by 14 mins/day for the control group. There was a significant group/time interaction effect.<br>Average daily sedentary time reduced in both groups (I: 4.8%, 55 mins; C: 2.9%, 34 mins) but there was no significant group/time interaction.<br>Fatigue also reduced in the intervention group and increased in the control group, with a significant group/time interaction effect.<br>Systolic blood pressure, gait speed, quality of life had significant time effect, but no group or interaction effect.<br>There were no significant results from other outcomes.

		creature being added to their fish tank e.g. sea horse or crab."	General Well-Being Index)	
Rosenberg, 2016[30]	Wearable activity trackers i.e. Fitbit Zip<br>*3.1 Social support (unspecified)*<br>*1.1 Goal setting (behavior)*<br>*2.3 Self-monitoring of behavior*	3.1 "Posting to social media sites", "creating networks with friends and family"<br>1.1 "setting goals"<br>2.3 "track step count, distance walked, and calories burned", "display visual presentations of data"	n/a	Thematic analysis revealed that most participants found the device comfortable and easy to wear; however, a barrier is technical problems i.e. perceived inaccuracy and sync problems. Participants were happy to share their PA data with HCPs, and they expressed a desire to go through their data with HCPs and get feedback.<br>Step count and distance walked were reportedly the most common feature used. Some participants used social features with family members. Very few participants reported using other Fitbit features (e.g. challenges, minutes active).
Middleweerd, 2015[27]	Nexercise app<br>*3.1 Social support (unspecified)*<br>*6.2 Social comparison*<br>*2.3 Self-monitoring of behavior*<br>*10.3 Non-specific award*	3.2 "chat features", "linking with social media"<br>6.2 "a competition feature"<br>2.3 "GPS tracking, activity log book"<br>10.3 "earning points"	n/a	Participants reportedly became more aware of their PA level through the app; however, they tended to stop using the app once the novelty disappeared and they encountered a technical problem. The preferred features included (1) goal setting, (2) self-monitoring and (3) a virtual coach that can motivate and provided tailored feedback towards personally set goals. Chat features were seen as redundant. The students also liked apps that enabled competition with friends or earning rewards. They would only share their PA data through social media only when the accomplishments were exceptionally positive.<br><br>There were some differences between people with high PA level and low PA level. Those with low PA level acknowledged that they liked getting Facebook likes for their achievements, and that it could make a difference to their behavior.
Pumper, 2015[29]	Facebook group<br>*3.1 Social support (unspecified)*<br>*10.6 Non-specific reward*<br>Fitbit Flex<br>*2.3 Self-monitoring of behavior*	3.1 "This group was a place where participants could ask questions, interact with both the moderator and the other participants"	n/a	Over the four-week intervention, on average, participants have 4.9 interactions in the form of likes (1.6 times), comments (0.6 times), and wall posts (0.3 times).

		10.6 "This group was a place where participants could […] receive weekly badges (i.e. virtual acknowledgements public to the group) for their fitness accomplishments"<br>2.3 "an activity tracker that can measure amounts of steps taking among other fitness measures"		Qualitative interviews revealed that the participants like being a part of the Facebook group as they perceived a sense of social support and membership, and the group also offered a comparison to their peers. Participants specifically liked the badge feature. They also reportedly tended to view the posts, but not contributed. They expressed the desire for more contribution to the group from both the other members and the moderator. They suggested that the moderator could give group members some ideas of what to post or included a motivational quote of the day.
Kernot, 2014[31]	Team-based Facebook group<br>*1.1 Goal setting (behavior)*<br>*2.2 Feedback on behavior*<br>*3.1 Social support (unspecified)*<br>*4.1 Instruction on how to perform the behavior*<br>*5.1 Information about health consequences*<br>*5.3 Information about social and environmental consequences*<br>*7.1 Prompts/cues*<br>*10.6 Non-specific reward*<br>Pedometer (NL-1000)<br>*2.3 Self-monitoring of behavior*	1.1 "used the app for 28 days with the cumulative goal being 280,000 steps"<br>2.2 "Additional feedback is provided regarding step count achievements via a team tally board, graphs", "Receive weekly emails detailing their progress"<br>3.1 "participated in teams of four to eight friends", "teammates can also send each other virtual gifts for encouragement"<br>4.1 "daily tips for increasing physical activity"<br>5.1, 5.3 "statistics on hours of life gained, fat burned, carbon emissions and transport costs saved"<br>7.1 "Receive weekly emails […] reminding them to log their steps"<br>10.6 "awards which participants can unlock based upon step count, login and team achievements"<br>2.3 "measured their daily step count with a pedometer"	n/a	Total activity time increased significantly by an average of 177 minutes/week. 68.4% of women accepted the invitation to join the Facebook team. Teams took a median of 13 days to form.<br>Facebook app was found to be easy to use, though participants reported difficulty finding the app on Apple devices and seeing all the features due to small screen size.<br>The average number of logins was 13.5 times throughout the 28-day intervention. There was a decline in log in rates towards the end of the study.
Al Ayubi, 2014[32]	Persuasive Social Network for Physical Activity (PersonA) mobile app<br>*1.1 Goal setting (behavior)*<br>*2.2 Feedback on behavior*<br>*2.3 Self-monitoring of behavior*<br>*3.1 Social support (unspecified)*<br>*6.2 Social comparison*	1.1 "allows users to define a target that they want to reach"<br>2.2 "Once the data is stored on the smartphone, it can be displayed as immediate and persuasive feedback", "visual feedback", "aural feedback"<br>2.3 "PA data to be captured automatically using sensor devices and then transferred to a smartphone", "self-monitoring chart […] shows how users can easily check the actual value for each activity item while they are performing a physical task. They can also monitor the progress they make by looking at the progress bar for each item and its percentage count, all of which is displayed on the same screen"	n/a	During the first week (app without social features), the step number/day increased by 4,202 on average. During the last three weeks (app with social feature), the mean step number/day increased by 6,352. Distance travelled increased by 1.15 miles per day in the first week, and by 1.74 miles per day in the last three weeks. No trends were apparent in the relationship between step number/day and social interaction. Overall, participants gave a score of 4.52 out of 5 for usability factors.

		3.1 “Third, the peer-support feature that allows individuals to support each other with one peer in a closed interaction where the individual and her/his peer only can see and communicate using this channel. Fourth, the group-support feature that allows users to support each other in open interaction where every member of the group can see and interact.”<br>6.2 “First, the peer-comparison feature allows an individual to compare his/her performance with that of one person in the app. This allows a more personal comparison, especially with a peer who is personally known, such as a close friend or spouse. Second, the group-comparison feature, which allows an individual to compare his/her current PA performance and target with the group average, the larger community average, or the normal standard set by health practitioners.”		Some participants said that they were not interested in social comparison as they had their own plan and schedule, while others found social comparison to be motivating and encouraging them to do more PA.
Khalil, 2013[33]	Step Up mobile app<br>*2.3 Self-monitoring of behavior*<br>*6.2 Social comparison*	2.3 “view number of steps walked, distance travelled, and calories burned”, “view walking history”, “view progress during the current week”<br>6.2 “view one’s team’s progress during the week”, “share step counts with their friends”	n/a	For the experimental study, during the second week, step counts increased for five out of seven participants. (Due to technical errors, data from one participant were removed.) The user survey indicated that the application was easy and fun to use. Six out of seven participants said using the app as a group motivated them to walk more. All seven participants said they liked to see friends’ steps, and that it motivated them to walk more. No one expressed concern about their friends’ ability to see their steps. Six participants reportedly tried to communicate with their friends when they noticed that one of their friends was not asking as much.

Abbreviation: BCTs: behavior change techniques; app: application; BMI: Body Mass Index (kg/m^2); n/a: not applicable; MVPA: moderate to vigorous physical activity; NR: not reported; SMS: short message services; PA: physical activity; OSN: online social network; ADHD: Attention deficit hyperactivity disorder; HCPs: health care providers; GPS: Global positioning system

Supplementary Information File 4

Behavior Change Techniques (BCTs) classification for non-experimental studies

First author, year	BCTs	Associated quotes
Stragier, 2016[36]	3.1 Social support (unspecified)<br>6.2 Social comparison<br><br>2.3 Self-monitoring of behavior	3.1 "users can interact with others[…]", "give kudos, […] equivalent of a Facebook like to activities posted by a Strava user, as a means of endorsing each other's achievement.", "comment on the activity"<br>6.1 "view other athletes' activities and can allow others to view theirs."<br>2.3 "manually add activities to their profile or to upload sessions logged through wearable devices or dedicated smartphone applications which use the sensors and GPS of the smartphone to automatically log a user's activities once a session is started."
Barlett, 2017[38]	**<u>Virtual coach system</u>**<br>3.1 Social support (unspecified)<br>1.1 Goal setting (behavior)<br>1.4 Action planning<br>2.2 Feedback on behavior<br>4.1 Instruction on how to perform the behavior<br>7.1 Prompts/cues<br>**<u>Music and maps system</u>**<br>1.1 Goal setting (behavior)<br>2.2 Feedback on behavior<br>2.3 Self-monitoring of behavior<br>7.1 Prompts/cues<br>**<u>Online community system</u>**<br>3.1 Social support (unspecified)<br>6.2 Social comparison<br>2.3 Self-monitoring of behavior<br>10.2 Material reward<br>10.3 Non-specific reward	3.1 "praise and encouragement from the virtual coach"<br>1.1, 1.4 "a suggested exercise plan with daily walking goals that increased to reach an overall goal (walking for 30 minutes)"<br>2.2 "recorded messages telling the user how many minutes they have been walking, or when they are halfway to their goal"<br>4.1 "Tips and advice on performing activity"<br>7.1 "choose to receive reminders to complete the activity"<br><br>1.1 "Set goals"<br>2.2 "feedback would be offered on a satellite map, as a summary table, or on a calendar (with activity levels shown for each day)"<br>2.3 "Track their activity using their mobile phone"<br>7.1 "local exercise facilities would be highlighted on the map"<br><br>3.1 "encourage interaction through […] collaborations"<br>6.1 "encourage interaction through competitions"<br>2.3 "track their activity using a mobile phone"<br>10.2 "real-world rewards (either through vouchers or donating money to charity)"<br>10.3 "points would be given when users achieved their goals (the details of the goal completed would not be shared", "earn virtual (stars or trophies on their profile) rewards"

Supplementary Information File 5

Risk of bias assessment for included randomized controlled trials[1]

Author, year	Random sequence allocation	Allocation concealment	Blinding of participants and personnel	Blinding of outcome assessment	Incomplete outcome data	Selective reporting
Ashton, 2017[20]	+	+	-	+	+	+
Mendoza, 2017[21]	+	?	-	?	+	?
King, 2016[22]	+	+	-	?	+	+
Greene, 2012[23]	+	?	-	-	-	?

+: Low risk of bias; -: High risk of bias; ?: Unclear risk of bias

[1] Assessment based on Cochrane's risk of bias tool

Supplementary Information File 6

Predefined search strategy

1.1 Search strategy for MEDLINE (via PubMed interface)
(https://www.ncbi.nlm.nih.gov/pubmed/)
Filters: none
Conducted in January 2018 and continuously updated until April 2018
#1 (((((((“Mobile applications”[MeSH] OR “Smartphone”[MeSH] OR tablet computer*[tiab] OR wearable device*[tiab] OR accelerometer*[tiab] OR activity monitor*[tiab] OR “Fitness trackers”[MeSH] OR fitbit*[tiab] OR armband*[tiab] OR arm band*[tiab] OR fitness watch*[tiab] OR pedomet*[tiab] OR wearable technolog*[tiab] OR wearable system*[tiab] OR wearable sensor*[tiab] OR fitness monitor*[tiab] OR garmin[tiab] OR bodymedia[tiab] OR nike fuelband[tiab] OR jawbone[tiab] OR step count[tiab] OR smartwatch*[tiab] OR smart watch*[tiab] OR sports watch*[tiab] OR wristband*[tiab] OR wrist band*[tiab] OR MyFitnessPal [tiab]))))
AND
#2 ((("Social Support"[Mesh] OR "Social Networking"[Mesh] OR “Reinforcement, Social”[Mesh] OR “Social media”[Mesh]) OR "social comparison" OR "social reward" OR "social network" OR "social influence" OR "social media" OR "social feature")))))

1.2 Search strategy for Embase
URL: Macquarie University Library (via OVID Interface)
Limits: none
Conducted in January 2018 and continuously updated until April 2018
#1 Mobile Application/ or Smartphone/ or pedometer/ or ("tablet computer" or "wearable device*" or "activity track*" or fitbit* or "fitness track*" or "fitness watch*" or "wearable system*" or "fitness monitor*" or garmin or bodymedia or "nike fuelband" or jawbone or "step count*" or smartwatch or "smart watch*" or "sports watch*" or wristband* or "wrist band*").mp
AND
#2 social support/ or social network/ or reinforcement/ or social media/ or ("social comparison" or "social reward" or "social network*" or "social influence" or "social media" or "social feature*").mp.

1.3 **Search strategy for PsycInfo**
URL: Macquarie University Library (via OVID Interface)
Limits: none
Conducted in January 2018 and continuously updated until April 2018
#1 Mobile Application/ or Smartphone/ or pedometer/ or ("tablet computer" or "wearable device*" or "activity track*" or fitbit* or "fitness track*" or "fitness watch*" or "wearable system*" or "fitness monitor*" or garmin or bodymedia or "nike fuelband" or jawbone or "step count*" or smartwatch or "smart watch*" or "sports watch*" or wristband* or "wrist band*").mp
AND
#2 social support/ or social network/ or reinforcement/ or social media/ or ("social comparison" or "social reward" or "social network*" or "social influence" or "social media" or "social feature*").mp.
